# Narrative review of magnetic resonance imaging in quantifying liver iron load

**DOI:** 10.3389/fmed.2024.1321513

**Published:** 2024-02-01

**Authors:** Qing Feng, Jixing Yi, Tao Li, Bumin Liang, Fengming Xu, Peng Peng

**Affiliations:** ^1^Department of Radiology, Fourth Affiliated Hospital of Guangxi Medical University, Liuzhou Workers' Hospital, Liuzhou, China; ^2^School of International Education, Guangxi Medical University, Nanning, China; ^3^Department of Radiology, The First Affiliated Hospital of Guangxi Medical University, Nanning, China

**Keywords:** magnetic resonance imaging, T_2_^*^, quantifying, liver, load of iron

## Abstract

**Objective:**

To summarize the research progress of magnetic resonance imaging (MRI) in quantifying liver iron load.

**Methods:**

To summarize the current status and progress of MRI technology in the quantitative study of liver iron load through reviewing the relevant literature at home and abroad.

**Results:**

Different MRI sequence examination techniques have formed a series of non-invasive methods for the examination of liver iron load. These techniques have important clinical significance in the imaging diagnosis of liver iron load. So far, the main MRI methods used to assess liver iron load are: signal intensity measurement method (signal intensity, SI) [signal intensity ratio (SIR) and difference in in-phase and out-of-phase signal intensity], T_2_/R_2_ measurement (such as FerriScan technique), ultra-short echo time (UTE) imaging technique, and susceptibility weighted imaging (including conventional susceptibility weighted imaging) (SWI), quantitative susceptibility mapping (QSM), T_2_^*^/R_2_^*^ measurement, Dixon and its derivative techniques.

**Conclusion:**

MRI has become the first choice for the non-invasive examination of liver iron overload, and it is helpful to improve the early detection of liver injury, liver fibrosis, liver cirrhosis and liver cancer caused by liver iron overload.

## 1 Introduction

As Liver is one of the main iron storage organs, and Liver iron concentration (LIC), that can reflect the total iron load, is used as an important clinical indicator for clinical monitoring, evaluation and treatment of iron overload (1).

Iron is an essential component of proteins in many important biochemical reactions, including hemoglobin, myoglobin, cytochromes, and peroxidases (2). Since the body has no natural mechanism for iron excretion, excess iron is stored in the liver (iron overload) (1, 2). As iron can promote the transformation of hydrogen peroxide into free radicals, excessive iron will produce toxicity, which can damage proteins, cell membranes and DNA, and it will eventually lead to organ damage (1). The causes of iron overload in human organs are different. The onset of patients with iron overload in organs is insidious, and the progression rate is different. The corresponding symptoms and signs are diverse and non-specific, and the degree of tissue and organ involvement is different. The diagnosis can often be made only when the organ is significantly damaged (3). Early and accurate diagnosis of organ iron overload is essential for timely treatment of patients and avoidance of irreversible organ damage (1, 3). The actual liver iron concentration provided by liver biopsy is often used as the “gold standard” for clinical quantitative iron content indicators, but biopsy is expensive and only provides small sample LIC. It possibaly may not reflect the overall liver LIC accurately, and has the shortcomings of invasiveness and poor repeatability. So it is not suitable for repeated longitudinal detection in treatment (1, 2). At present, most scholars and medical centers prefer to use non-invasive magnetic resonance imaging (MRI) technology for quantitative evaluation of LIC and monitoring of liver iron chelation efficacy (1, 3–5). With the continuous development of MRI equipment and imaging sequences, T_2_^*^ technique based on MRI gradient recalled echo (GRE) imaging sequence has been identified as the non-invasive preferred method for quantifying tissue iron content (2, 4–6). Many centers have been using the T_2_^*^ relaxation method, the corresponding calibration formula and different software techniques to measure the relevant relaxation parameters of organs, such as T_2_^*^ and R_2_^*^ (1,000/T_2_^*^) values, so as to indirectly obtain the estimates of LIC of organs (4, 6). Currently, accurate quantification of organ iron content remains challenging, especially in patients with severe organ iron overload (1, 4, 6). Therefore, scholars are still looking for a reliable, noninvasive, accurate and reproducible assessment method of organ iron overload (3). At present, the methods used to detect LIC mainly include: Liver biopsy, laboratory tests such as serum ferritin (SF) and transferrin saturation (transferrin saturation, TS) detection, superconducting quantum electromagnetic interference (SQUID) iron quantification, computed tomography (CT) and MRI related technologies. This article reviews the basic principles, research progress and application status of magnetic resonance imaging T_2_^*^ technology and other related magnetic resonance imaging techniques for quantifying liver iron load.

## 2 Liver tissue biopsy

As mentioned above, LIC reported by liver biopsy have long been used as the “gold standard” for clinical quantification of liver LIC (1, 3–5). However, due to different research centers or medical institutions, some factors may lead to discrepancies between LIC reported by liver biopsy, including the materials and methods used in the process of liver biopsy, and the heterogeneity of iron in liver tissue, etc. Some studies have pointed out that early liver iron deposition is uneven and irregular (7, 8), and the “spot sampling” of liver biopsy may not reflect the overall liver iron deposition. Moreover, repeated sampling and long-term LIC monitoring in patients with liver iron overload are not recommended because of the invasiveness and risk of complications of this method (1, 6). Therefore, liver biopsy is not an ideal method for long-term assessment of liver iron burden and monitoring the efficacy of iron chelation therapy.

## 3 Laboratory tests

Two measurement indexes commonly used in the laboratory to evaluate the iron load of organs in patients are SF and TS (3). Although SF and TS can reflect the iron load in human blood to a certain extent, they have no obvious correlation with organ iron deposition. The degree of correlation is not high enough to accurately quantify the iron content of liver or other organs (6, 8). Moreover, it is not comprehensive to only use SF and TS as indicators of iron load in human body. Because these two indicators are likely to show large changes due to inflammation, infection, blood transfusion, or other chronic diseases, SF will be too low even when patients have severe iron overload in organs (9, 10).

## 4 SQUID detection

Superconducting quantum interference device (SQUID) is a highly sensitive magnetic field detection instrument, which can measure the magnetic susceptibility of liver or other organs in a non-invasive way. Then the iron content of liver or other organs was quantitatively evaluated (11). It has been experimentally verified that the results of SQUID quantification of liver iron content show a good correlation with LIC reported by liver biopsy (10). However, the high cost of using this device and its very limited availability are the biggest limitations for the use of this device to quantify the iron content of organs. And the requirement of professionals for data measurement collection and equipment maintenance also limits its widespread use (3, 12). Therefore, SQUid-based measurements are currently not widely used in clinical practice for long-term quantitative monitoring of LIC in patients with iron overload.

## 5 CT examination

It is proved that X-rays decay proportionally with increasing tissue iron concentration. In the absence of intravenous contrast agent injection, if the liver density CT measurement value is ≥75HU, liver iron overload can be suspected (3). Although CT may be able to qualitatively monitor and assess liver iron overload in patients, attenuation or increase in liver CT measurements is not entirely due to iron overload (13). Recently, some studies have shown that dual-energy CT imaging can achieve quantitative assessment of LIC even in the presence of liver steatosis, and it has shown good correlation and consistency (14, 15). However, in addition to hepatic steatosis, other confounding factors, including Wilson’s disease, glycogen deposition, and certain medications (e.g., amiodarone), may also alter liver CT measurements (13, 14). Because ionizing effects can cause radiation damage to patients, CT examination is not the best choice for repeated measurement of LIC during iron chelation therapy monitoring in patients with liver iron overload.

## 6 MRI examination

In recent years, with the development of new MRI imaging techniques, more and more MRI imaging techniques have been applied to quantitative or semi-quantitative assessment of liver iron load in patients with iron overload (1, 4). Key approaches include: signal intensity (SI) measurement [including signal intensity ratio (SIR) and the difference of signal intensity in the same and opposite phase], T_2_/R_2_ measurement (such as FerriScan technique), T_2_^*^/R_2_^*^ measurement, ultroshort echo time (ultroshort echo time, UTE), Dixon and its derivative techniques, and susceptibility weighted imaging techniques [conventional susceptibility weighted imaging (SWI) and quantitative susceptibility mapping (QSM)]. These techniques can quantify the organ iron load and reflect the severity of organ iron overload in patients with iron overload on the basis of non-invasiveness and no radiation damage, and the efficacy of iron chelation therapy can be evaluated in patients with iron overload (16, 17). Therefore, quantitative MRI imaging technology plays a very important role in the diagnosis, clinical classification, severity assessment and efficacy monitoring of iron chelation in liver iron overload. The merits and faults of different MRI examination methods are shown in Table 1.

**Table 1:** Merits and faults of different MRI methods in quantitative LIC.

Method	Merits	Faults
SI (signal intensity)	Can reduce the error caused by different equipment and magnetic field inequality, and make the detection and evaluation of LIC more accurate	Not suitable for quantification of LIC higher than 19.5 mg /g (350 μmol/g)
FerriScan (R_2_ Relaxometry-FerriScan^®^)	After multi-center verification and continuous high-quality data, it has a relatively stable calibration curve, and it is not easily affected by many factors. (Usually used as a non-invasive reference standard for assessing LIC)	It takes a long time (The MRI scan time and the LIC analysis time were included) Patient data will need to be sent out Generates additional outgoing analytical sub-costs
T_2_^*^/R_2_^*^ relaxation measurement	A fast-scanning MRI technique (A breath) T_2_^*^/R_2_^*^ and LIC show a very good linear correlation, excellent consistency and reproducibility The special “cut-off method” can improve the goodness of fit of the measured T_2_^*^	The LIC quantitative analysis with different MRI scan sequence parameters and different image analysis software has always been considered as a limitation The upper limit of LIC for high field strength MRI is small (the upper limit of LIC detection at 3 T is 26 mg / g dry weight)
Dixon and its derivative technology	Can effectively correct the inhomogeneity of the magnetic field and the error caused by the T_2_^*^ attenuation By breath-holding, water, fat and fat ratio images were obtained to eliminate the effect of T_2_^*^ on fat content The liver can be quickly detected for iron overload, and if combined with steatosis	Further research is still needed in the accurate quantitative LIC
SWI (susceptibility weighted imaging)	It can improve the detection rate of mild hepatic iron deposition and have higher sensitivity for the diagnosis of cirrhosis and iron-containing nodules	This technique is based on the quantitative measurement of the tissue signal value and only the semi-quantitative measurement of the corresponding iron content The conventional SWI has a geometric dependency
QSM (quantitative susceptibility mapping)	The combination of QSM and SWI can avoid the geometric dependence of conventional SWI, and more accurately show the material and structural organization with high magnetic sensitivity	Despite a high correlation between the measured susceptibility and R_2_^*^, performing a conversion between multiple measured parameters may have an impact on the final LIC assessment

### 6.1 SI measurement method

Based on the correlation between LIC and its signal intensity, SI measurement method (mainly including SIR And difference in signal intensity of in-phase and out-of-phase) is used for semi-quantitative diagnosis of iron overload by measuring and calculating the ratio of SI of the liver and paraspinal muscles (such as erector spinae) without iron at the same level of the liver (3, 18). By reflecting the complex nonlinear relationship between LIC and SI, SIR Measurement can be used to assess the iron load in patients with moderate liver iron overload (18–20). Its advantage is that it can reduce the errors caused by different equipment and magnetic field heterogeneity, so that LIC detection and evaluation are more accurate (19, 20). The original SIR Method is suitable for iron overload of low to high severity, but not for LIC quantification above 19.5 mg/g (350 μmol/g) (1). Early research data from Ernst et al. (18) showed that the range of liver iron concentration that SIR could detect was 50–300 μmol/g, but it had low accuracy in detecting liver iron overload less than 50 μmol/g or more than 300 μmol/g. The SIR Method has been verified at 1.5 T and 3 T, but there is no regulatory approval at present (1). However, d'Assignies et al. (19) suggested that the use of 3 T MRI SIR may be able to more accurately quantify the liver iron load in patients with severe liver iron overload. The wider measurement range of the SIR Measurement may be due to the lower sensitivity of the spinecho (SE) sequence to iron. And it leads to delayed signal loss at high LIC due to the shorter TE, thus allowing the assessment of more severe liver iron overload. In recent years, Jensen et al. (21) found that the upper limit of 1.5 T MRI SIR Measurement range would be extended to 115 mg/g if the relevant parameters TE = 12 ms and TR = 1,200 ms were set in the liver iron overload experiment of quantitative miniature pigs. However, this conclusion was drawn in an animal model and requires further validation in iron-loaded patients. Therefore, the current SIR Method for measuring liver iron overload should be considered as an alternative to R_2_ and R_2_^*^ relaxation quantification methods.

### 6.2 Relaxation measurement method

At present, the main MRI sequences used to measure LIC are SE sequence and GRE sequence. With SE and GRE sequences, based on the influence of iron, the transverse relaxation of proton magnetization in water becomes faster, resulting in attenuation of magnetic resonance signal intensity, and T_2_ and T_2_^*^ weighted imaging are obtained, respectively. Then the corresponding signal decay time constants T_2_ and T_2_^*^ and the corresponding relaxation rates R_2_ (1,000/T_2_) and R_2_^*^ (1,000/T_2_^*^) were obtained (1, 3, 22). Relaxometry is a quantitative assessment of MRI signal loss caused by the shortening of T_2_/T_2_^*^ relaxation times (21, 22). Excess iron stored in the body in the form of trivalent iron can shorten T1 and T_2_—as T1 shortens, the corresponding SI increases; The corresponding SI decreases with the shortening of T_2_ (1, 3).

#### 6.2.1 T_2_/R_2_ relaxation measurement method

The T_2_/R_2_ relaxation measurement method is based on the T_2_ SE sequence (a time-wasting sequence whose transverse relaxation time depends on the iron content of the tissue) to evaluate the iron concentration in different tissues by measuring the size of the T_2_ value (23). The R_2_ relaxation quantitative method is based on SE signals of multiple TE, and the attenuation of R_2_ in this method is mainly composed of irreversible spin echo R_2_ (1, 3, 23). St Pierre et al. (24) obtained the calibration constant of the single exponential attenuation model by analyzing specific MRI imaging parameters. This method enables LIC conversion of the obtained R_2_ measurements, and this quasi-method is called R_2_ Relaxometry^®^ (FerriScan) (24). FerriScan is a widely validated 1.5 T MRI technique that has been certified and approved by the Food and Drug Administration (FDA) for commercial use for safe, reliable, and noninvasive LIC assessment. Quantification of a wide range of LIC can be achieved based on five T_2_-weighted SE sequence acquisitions during free breathing, with TE added to calculate R_2_ (1, 24, 25). The R_2_-Ferriscan method has a relatively stable calibration curve due to multi-center validation and continuous high-quality data, and is not affected by multiple factors (MRI equipment, patient age, fibrosis stage, inflammation, iron chelator treatment, etc.). It is commonly used as a noninvasive reference standard for evaluating LIC (1, 24). However, this technology also has many limiting problems: (1) This method takes a long time to collect, patients may feel uncomfortable or anxious, and errors will be introduced by movement (1); (2) Because T_2_/R_2_ sequence is not easy to measure fat, one of the limitations of FerriScan is that it cannot quantify the fat content in liver tissue (26). (3) The MRI T_2_/R_2_ data of the patients were sent to FerriScan for offsite post-processing and analysis. However, sending patient data off-site requires the approval of the relevant center, and the time cost required will prolong the time of obtaining LIC results. (4) The additional analysis cost will increase the monitoring cost of LIC. These factors concurred to the fact that liver iron quantification using the FerriScan technique is limited to a few large medical centers or research institutions, and the possibility of monitoring patients with LIC on a regular or long-term basis is substantially reduced (24, 27, 28).

#### 6.2.2 T_2_^*^/R_2_^*^ relaxation measurement method

T_2_^*^/R_2_^*^ relaxation measurement is a fast scanning MRI technique that can obtain the corresponding T_2_^*^ image data only after a patient holds his or her breath once. At present, many studies have shown that T_2_^*^/R_2_^*^ relaxation quantitative methods have good linear correlation when measuring LIC at 1.5 T and 3 T. T_2_^*^ is negatively correlated with LIC. R_2_^*^ is positively correlated with LIC and has shown excellent agreement and reproducibility (1, 4, 5, 22, 23, 27, 29), and this measurement has become a reliable quantitative assessment of liver iron overload. Figure 1 from Xu et al. (4) shows liver T_2_^*^ measured by different software. There are two other free software from Prof Gandon in France (19):

(1) https://imagemed.univ-rennes1.fr/en/mrquantif/quantif
(2) http://www.isodense.com/ic/

**Figure 1:**
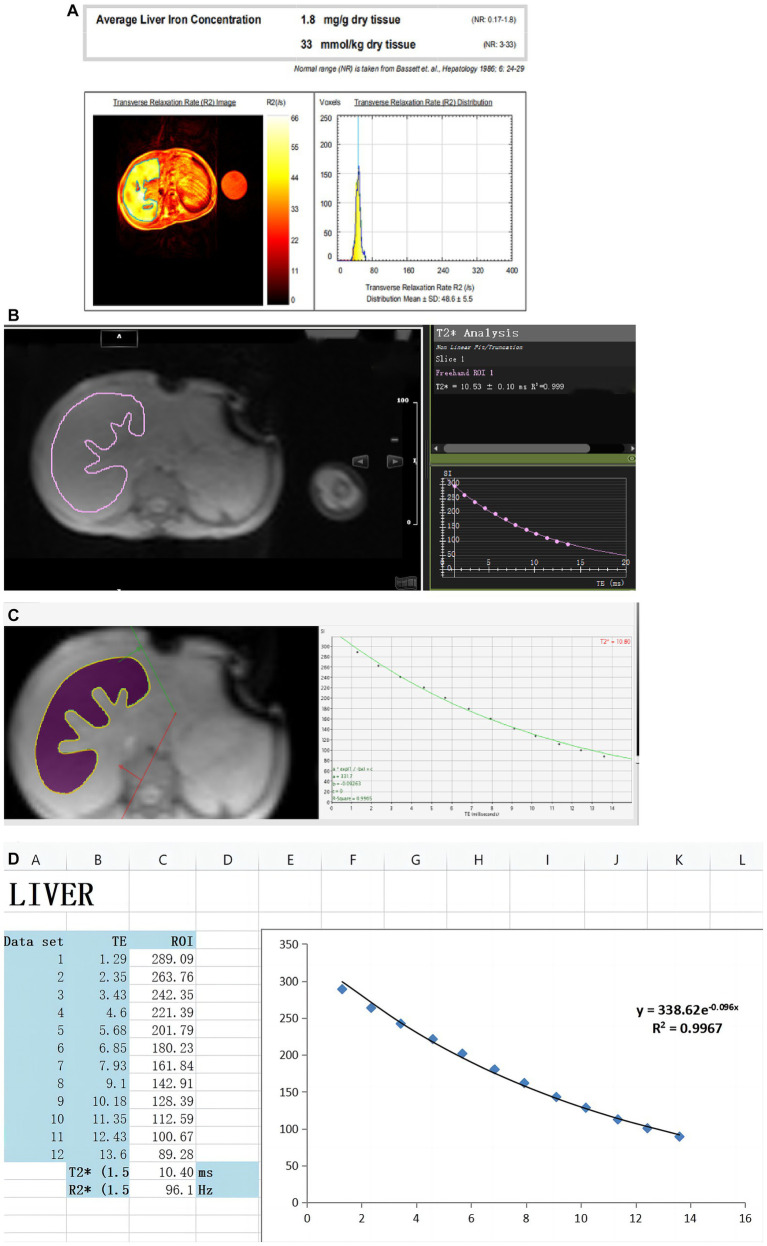
Created by Xu et al. (4) can be reused under the CC BY license (https://creativecommons.org/licenses/by-nc/4.0). These figures show LIC was quantified in the same thalassemia patient using different software. **(A)** Report from the FerriScan: LIC = 1.8 mg/g dw. **(B)** Report from Circle Cardiovascular Imaging CVI42 (CVI42): T_2_^*^ = 10.53 ms. **(C)** Report from CMRtools/Thalassemia Tools (CMRtools): T_2_^*^ = 10.80 ms. **(D)** Report from Excel spreadsheet (Excel): T_2_^*^ = 10.40 ms.

However, the different MRI scanning sequence parameters and image analysis software used in many studies have been considered as a limitation (26, 30, 31). Studies have shown that the existing bias can be corrected, and in some cases, the Goodness of Fit (R^2^) of the measurement can be improved by using the truncation method to remove part of the interfering signal that affects the background noise. It thereby provides clinically acceptable LIC estimation and reproducible results (31). Therefore, many medical centers or scientific research institutions have been using T_2_^*^/R_2_^*^ relaxometry, self-made MRI sequences and corresponding post-processing software for quantitative assessment of liver iron overload (5, 30, 32–38). Using the R_2_^*^ relaxation method in the quantitative study of liver iron overload, Henninger et al. (6) initially performed liver biopsy and MRI in 17 patients with clinically suspected liver iron overload with the relevant parameters set as repetition time (TR) = 200 ms and initial echo time (TE) = 0.99 ms. Finally, the regression model between R_2_^*^ and LIC was constructed as follows. LIC = 0.024R_2_^*^ + 0.277, correlation coefficient = 0.926, slope = 0.024 (mg/g) [95%CI = 0.013–0.024], intercept = 0.277 (mg/g) [95%CI = 0.328–2.49]. In an early study by Wood et al. (30), the set TE was increased from the initial 0.8–4.8 ms at an interval of 0.25 ms in a breath-hold, with TR = 25 ms. After MRI evaluation of 102 patients with liver iron overload (the biopsimeasured LIC was evenly distributed between 1.3 mg/gdw and 32.9 mg/gdw, and one patient had a HIC of 57.8 mg/g dw), the final LIC-R_2_^*^ regression equation was constructed as follows: The correlation coefficient was 0.97, the slope was 37.4 Hz/mg/gdw, and the y-intercept was 23.7 Hz. In the early study of Hankins et al. (33), TE = 1.1–17.3 ms (20 echoes) was set, and 43 patients (32 with sickle cell anemia, 6 with major β-thalassemia, 6 with mild thalassemia) were tested. Five patients with bone marrow failure underwent MRI examination and liver biopsy (LIC range = 0.6 mg Fe/g to 27.6 mg Fe/g). The final LIC-R_2_^*^ regression model was constructed as follows: The intercept was −454.85, the slope was 28.02 (*p* < 0.001), the R_2_ was 0.72, and the correlation coefficient was 0.98. In an early study by Christoforidis et al. (34), MRI was performed on 94 patients with β-thalassemia major with TE = 2.24 ms ~ 20.13 ms and TR = 200 ms. By comparing the relationship between liver-muscle ratio (MRI-LIC = 5 μmol/g ~ 350 μmol/g) and R_2_^*^ (27.03 s^−1^ ~ 1298.70 s^−1^), the final LCI-R_2_^*^ regression model was constructed as follows: R_2_^*^ = 0.851(MR-LIC) - 2.137 (correlation coefficient = 0.851). In the study of Garbowski et al. (35), TE = 0.93 ms ~ 16.0 ms was set. Fifty-four patients (36 cases of thalassemia major, 7 cases of sickle cell anemia, 4 cases of myelodysplastic syndrome, 3 cases of Diamond-Blackfan anemia, 2 cases of red cell aplasia, 2 cases of pyruvate kinase deficiency anemia) and 31 healthy volunteers underwent liver biopsy (LIC = 1.7 mg/g dw ~ 42.3 mg/g dw) and MRI (R_2_^*^ range: 28.7 s^−1^ to 54.4 s^−1^). Finally, the regression models of LIC (biopsy)-T_2_^*^ and LIC (biopsy)-R_2_^*^ were constructed: (1) LIC = 31.94(T_2_^*^)^−1.014^, 95%CI of coefficient = 27.8 ~ 36.7 (87% ~ 115%), 95%CI of index = −1.118 ~ −0.91 (110% ~ 90%). (2) LIC = 0.029R_2_^*1.014^, 95%CI of coefficient = 0.016 ~ 0.054 (55% ~ 186%), 95%CI of index = 0.910 ~ 1.118 (90% ~ 110%). Garbowski et al. also constructed the correction relationship between LIC(Ferriscan)-R_2_^*^ and LIC(T_2_^*^)-T_2_^*^: (1) R_2_-LIC = 0.83 T_2_^*^-LIC^1.04^, 95%CI of coefficient = 0.96 ~ 1.11, 95%CI of index = 0.55 ~ 1.29. (2) R_2_-LIC = 0.87R_2_^*^-LIC -0.55, 95%CI of slope = 0.74 ~ 0.99, 95%CI of intercept = −0.01 ~ 1.19. The linear relationship between the relaxation parameters and the LIC for the different studies is shown in Table 2.

**Table 2:** The linear relationship between the relaxation parameters and the LIC.

Scholar	Reference standards	Linear formulas
Henninger	Liver biopsies were performed in 17 patients with clinically suspected liver iron overload	LIC (mg/g dw) = 0.024R_2_^*^(s^−1^) + 0.277
Wood	21 liver biwere taken (biopsy measured LIC was evenly distributed between 1.3 mg/g dw and 32.9 mg/g dw and 1 patient with an HIC of 57.8 mg/g dw)	LIC (mg/g dw) = 0.0254R_2_^*^(s^−1^) + 0.202
Hankins	MRI and liver biopsy were performed in 43 patients (LIC range = 0.6 mg Fe/g to 27.6 mg Fe/g)	LIC (mg/g) = 0.028R_2_^*^(s^−1^) - 0.45
Christoforidis	MRI was performed in 94 patients with severe β-thalassemia, comparing the relationship between liver-muscle ratio (MRI-LIC = 5 μmol/g ~ 350 μmol/g) and R_2_^*^ (27.03 s^−1^ ~ 1298.70s^−1^)	R_2_^*^(s^−1^) = 0.851[MR-LIC (μmol/g)] - 2.137
Garbowski	Liver biopsies were performed in 54 patients and 31 healthy volunteers (LIC range = 1.7 mg/g dw ~ 42.3 mg/g dw) and MRI (R_2_^*^ range = 28.7 s^−1^ ~ 54.4 s^−1^)	LIC (mg/g) = 0.029R_2_^*1.014^

With the development of The Times, high-magnetic field MRI (3 T and above) scanners may gradually replace low-magnetic field MRI scanners because of their high contrast images (30, 33). However, T_2_^*^/R_2_^*^ relaxation measurement method has a certain range for LIC measurement: [The upper limit of LIC detection is 26 mg/g (466 μmol/g) at 3 T, and 52 mg/g (932 μmol/g) at 1.5 T] (1). Moreover, with the increase of magnetic field, the decrease rate of T_2_^*^ value is faster, which may make a big difference in the accurate quantitative analysis of liver iron load in patients with iron overload in higher magnetic field. Although previous studies have reported that the high sensitivity of 3 T MRI for liver iron quantification in patients with iron overload can more accurately analyze and detect mild iron overload, some studies have used 3 T MRI T_2_^*^/R_2_^*^ technology to quantify liver iron overload only for the diagnosis of iron overload. However, it is not possible to accurately quantify LIC in patients with moderate to severe liver iron overload at 3 T field strength, especially those with LIC > 26 mg/g (466 μmol/g). It is strongly recommended that 3 T T_2_^*^ method should be avoided to quantify LIC in patients with severe iron overload. Whereas 1.5 T or other methods are used (1, 18, 19).

#### 6.2.3 Dixon and its derivative technology

Based on the fact that water and fat have different precession frequencies in the magnetic field, the in-and out-phase of water and fat can be obtained by adjusting the Dixon technique of chemical shift imaging of TE (3, 23). Then by computational processing, images of separate water or fat signals can be obtained (3, 23). As a T_2_^*^-weighted sequence examination method capable of quantifying fat, Dixon technique is mainly used in fat quantification studies of the liver (3, 36, 37). Meanwhile, R_2_^*^ mapping obtained from T_2_^*^ using this technique can also be used for quantitative analysis of liver iron overload (36, 37). Since the development of Dixon technique, its related imaging techniques have been continuously improved, from the initial acquisition of signals under two echoes (two-point acquisition) to the acquisition of signals under three or six echoes.

The disadvantage of two-point Dixon imaging is that it is easy to be affected by the interference of the non-uniform main magnetic field and the attenuation effect of T1 and T_2_^*^. It will lead to fuzzy display of the structure of the interface between water and fat on the image, resulting in incomplete separation of water and fat (34–39). Three-point Dixon water-fat separation imaging technology can overcome the above shortcomings: Three-point Dixon imaging is characterized by the acquisition of an echo signal in the same phase on the basis of two-point imaging. The acquisition time of the middle signal is the same as that of the spin echo/fast spin echo (FSE) sequence. It can correct the rapid decay of T_2_^*^ to a certain extent (34, 35, 38). However, because the liver fat fraction obtained by three-point Dixon imaging is also susceptible to various confounding factors, the accuracy, reliability and repeatability of its results as liver fat quantification still need further study (40).

At present, chemical replacement water-fat separation imaging technology with multiple gradient echoes is the most common, such as the Liver Laboratory Liver-LabqDixon and IDEAL-IQ technology from Siemens. Compared with the early Dixon technique, the six-echo Dixon technique can effectively correct the magnetic field inhomogeneity and the error caused by T_2_^*^ attenuation, and make the measurement result more accurate. DEAL-IQ achieves dynamic 0 to 100% fat ratios by reconstructing complex domains. By holding the breath, water, fat and fat ratio images were obtained and the effect of T_2_^*^ on fat content can be eliminated. Fat distribution map can not only directly measure fat content, but also reflect fat distribution (41). This method can be applied to the measurement and analysis of visceral fat content, the application of fat quantification technology in musculoskeletal system diseases, and it also can be applied to the quantitative measurement of iron, such as the detection of iron deposition in the central nervous system of patients with Alzheimer’s disease and Parkinson’s disease, and the quantitative analysis of iron overload in solid organs and endocrine glands. It is not limited in the test of organs, and can be used in the test of heart, liver, pancreas and spleen (41, 42). At present, most published studies on iron overload at home and abroad are mainly based on IDEAL-IQ technology (42). LAVA-Flex sequence is a 3D disturbed gradient echo sequence based on Dixon technique. This technique is to obtain pure water image, pure fat image, in-phase image and out-of-phase image by applying machine to post-process the original data (42, 43). Then it is combined to determine whether the liver has iron overload, and LAVA-Flex sequence can be used to quickly detect whether the liver has iron overload and whether it is complicated with steatosis (43). Dixon technique has a very optimistic application prospect either as a means of scientific research at present or as an independent clinical detection project in the future.

### 6.3 Susceptibility weighted imaging and QSM techniques

Susceptibility weighted imaging (SWI), based on T_2_^*^-weighted gradient echo sequence, provides image contrast enhancement according to the difference in magnetic sensitivity between different tissues. It is an imaging technique that can obtain a phase image and a magnitude image at the same time. The basic principle of this technology is to perform high-resolution 3D gradient echo imaging based on T_2_^*^ -weighted gradient echo sequence to detect the difference in magnetic sensitivity between different tissues for comparative analysis (44).

SWI has become a widely used imaging diagnostic technique in clinical practice, which is often used for the differential diagnosis of cerebral hemorrhage, intracerebral microvascular hemorrhage and intracranial calcification. This technique is based on quantitative measurement of tissue signal values and semi-quantitative measurement of corresponding iron content, which can improve its sensitivity to iron (3, 22, 45). With the continuous application of MRI technology in the detection of iron overload, the application of SWI in the study of liver iron overload is also increasing. Compared with other MRI imaging sequences such as T_2_ SE and T_2_^*^ gradient echo, SWI has some different advantages: SWI can improve the detection rate of mild hepatic iron deposition, and has higher sensitivity for the diagnosis of small iron-containing nodules in liver cirrhosis (45, 46).

Although SWI has made progress in the related research of liver iron overload, the current conventional SWI has geometric dependence. The quantitative assessment of liver iron burden needs further in-depth research (44). QSM can reduce phase confounding and the limitation of T_2_ signal attenuation by using short TE. Moreover, the combination of SWI and QSM can avoid the geometric dependence of conventional SWI, and can more accurately display substances and structures with high magnetic sensitivity (44, 46).

At present, studies based on abdominal QSM technology have verified the feasibility of QSM technology in quantitative detection of liver iron in patients with iron overload (47). Sharma et al. (48) confirmed that quantitative susceptibility mapping-based biomagnetic liver susceptometry (QSM-BLS) can provide clear three-dimensional images. Moreover, the magnetic susceptibility measured by QSM technology has a high correlation with R_2_^*^, which can be used to correct R_2_^*^ and evaluate liver iron load, especially when SQUID equipment is lacking for accurate quantification (44–47).

## 7 Conclusion

In summary, although there are many methods to detect liver iron load, MRI has become an important method in clinical practice to detect liver iron load in patients with iron overload due to its advantages of non-invasiveness, accuracy and repeatability (3, 5). This method is helpful to improve the early detection of liver damage, liver fibrosis, cirrhosis and even liver cancer in patients with iron overload. However, most of the existing studies based on the effect of MRI on the detection of liver iron concentration have a certain quantitative range, and the quantitative analysis of liver iron concentration in patients with iron overload by high-field MRI is limited. It is necessary to further optimize the MRI sequence and establish a perfect and standardized data analysis method. This will further improve the clinical application of MRI in the diagnosis of liver iron overload and monitoring the efficacy of iron chelation therapy.

## Author contributions

QF: Writing – original draft, Writing – review & editing. JY: Writing – original draft, Writing – review & editing. TL: Writing – original draft, Writing – review & editing. BL: Writing – original draft, Writing – review & editing. FX: Writing – original draft, Writing – review & editing. PP: Funding acquisition, Writing – review & editing.
